# <i>Aedes aegypti</i> Control Through Modernized, Integrated Vector Management

**DOI:** 10.1371/currents.outbreaks.45deb8e03a438c4d088afb4fafae8747

**Published:** 2017-01-30

**Authors:** Laith Yakob, Sebastian Funk, Anton Camacho, Oliver Brady, W. John Edmunds

**Affiliations:** Department of Disease Control, The London School of Hygiene & Tropical Medicine, London, United Kingdom; Department of Infectious Disease Epidemiology, The London School of Hygiene & Tropical Medicine, London, United Kingdom; Department of Infectious Disease Epidemiology, The London School of Hygiene & Tropical Medicine, London, United Kingdom; Department of Infectious Disease Epidemiology, The London School of Hygiene & Tropical Medicine, London, United Kingdom; Department of Infectious Disease Epidemiology, The London School of Hygiene & Tropical Medicine, London, United Kingdom

## Abstract

Introduction: In the context of the ongoing, unprecedented Zika virus outbreak in the Americas, the World Health Organization has expressed its support for developing and up-scaling three novel approaches to controlling the *Aedes aegypti* mosquito: the Sterile Insect Technique (SIT), the Release of Insects carrying Dominant Lethal genes (RIDL) and the release of *Wolbachia*-infected mosquitoes. Whereas the former two approaches are temporary insect population suppression strategies, *Wolbachia* infection is a self-sustaining, invasive strategy that uses inherited endosymbiotic bacteria to render natural mosquito populations arbovirus resistant.

Methods: A mathematical model is parameterised with new, Brazilian field data informing the mating competitiveness of mass-reared, released insects; and simulations compare and contrast projections of vector control achieved with the alternative approaches.

Results: Important disadvantages of *Wolbachia* and SIT are identified: both strategies result in mosquitoes ovipositing non-viable eggs and, by alleviating intense larval competition, can cause an overall increase in survival to the adult stage. However, it is demonstrated that strategically combining the suppression methods with *Wolbachia* can generate a sustained control while mitigating the risks of inadvertent exacerbation of the wild mosquito population.

Discussion: This initial analysis demonstrates potential for good synergy when combining novel mosquito approaches in a modernized, integrated vector control programme.

## Introduction

Autochthonous American transmission of Zika virus was first identified in May 2015 following its isolation from febrile individuals from the Brazilian states of Bahia and Rio Grande do Norte. In the subsequent 15 months, confirmed cases of ZIKV had been declared by health authorities of over 50 countries and territories in the region^1^ and estimates of the number of suspected cases for 2015 alone exceed 1 million.^2^

ZIKV is transmitted through the bite of several mosquito species, but *Aedes aegypti* (the primary dengue and chikungunya vector) is believed responsible for the current surge. Although the virus was discovered to infect humans in 1952,^3^ it was not until recent months, in the wake of an outbreak of unprecedented size, that a possible link between infection during pregnancy and subsequent neurological disorders (including microcephaly) in newborns was first made.^2^ On February 1st 2016, this association led the World Health Organization to declare the American Zika virus outbreak a public health emergency of international concern (PHEIC).

Epidemiological understanding of ZIKV is limited. Prior to a 2007 outbreak on Yap Island^4^ and a 2013-14 outbreak in French Polynesia,^5^ the virus was believed to be zoonotic, chiefly infecting monkeys and only yielding the occasional spillover into small numbers of humans. Consequently, few empirical studies exist to inform many basic epidemiological metrics such as the incubation periods in mosquitoes and humans; the rate of symptomatic infection; the duration of infectivity of mammalian hosts or the development of immunity to infection. A huge, concerted effort among the international research community following the WHO declaration means that the coming months and years can be expected to steadily populate these important knowledge gaps. Corresponding improvements in case management and prophylaxis can also be anticipated. Until then, however, vector control is the only available strategy to mitigate further outbreaks and contain the spread of disease.^6^

Due to its primary role in the spread of major arboviral public health threats including dengue, chikungunya and yellow fever, the vector *A. aegypti* has been the target of numerous novel vector control technologies generated in recent decades. These include methods that either suppress mosquito popuations or render mosquitoes refractory to arboviral infection.^7^ The Sterile Insect Technique (SIT) is a genetics-based method that has been successfully used to suppress agricultural pest insects since the 1950s.^8^ It involves the release of radiation-sterilised male insects into wild populations where they seek out and mate with the females, giving rise to offspring which are not viable. A limitation of this method that has so far hampered its implementation in mosquito control is the considerable reduction in mating competitiveness incurred in these insects through exposure to radiation.^9^

The Release of Insects carrying a Dominant Lethal gene (RIDL) is a strategy related to SIT but with a dominant lethal transgene inserted into the mosquito replacing the need for radiation exposure.^10^ An important distinction between a trangenic lethal gene and radiation sterilisation is the ability within the former approach to control the timing of lethal gene activation. Whereas SIT offspring perish at the insect’s egg stage, RIDL can be programmed to take effect at any life stage. A line of RIDL *A. aegypti* mosquitoes that express the lethal gene at the pupal stage was recently described.^11^ Surviving beyond the egg stage to die as pupae may be advantageous because RIDL larvae will survive to compete with wild larvae for food, providing a secondary mode of control that operates through enhancing resource limitation. A small-scale field trial of RIDL in the Brazilian state of Bahia was recently conducted and reported to have a considerable level of success in suppressing wild *A. aegypti* populations.^12^

The third approach to vector control that is considered in the current study is the release of *Wolbachia*-infected male mosquitoes. *Wolbachia* are endosymbiotic bacteria that have recently been demonstrated to render* A. aegypti* mosquitoes resistant to infection with dengue^13^ and chikungunya;^14^ and there is some early indication that *Wolbachia*-infected *A. aegypti* are also refractory to Zika virus.^15^ *Wolbachia* infection produces a phenotype termed cytoplamic incompatability whereby infected female insects have viable offspring but uninfected females do not have viable offspring when they mate with infected males. This results in a selective advantage from infection that facilitates the spread of *Wolbachia* into wild insect populations. The first field trial was conducted in northern Queensland, Australia, where repeated releases of *Wolbachia* infected male *A. aegypti* resulted in the successful establishment of the endosymbiont in the local wild mosquito populations.^16^

The two suppression methods, SIT and RIDL, along with the arbovirus refractory-method employing *Wolbachia*, have all been endorsed by the WHO to be used for vector control in the current Zika PHEIC.^17^ Here, a mathematical model is presented that i) assesses the relative efficacies projected for these novel vector control methods, ii) identifies any anticipated shortcomings of the different approaches, and finally, iii) strategises their combined implementation as part of a modernized, integrated vector management programme.

## Methods

***Aedes aegypti* population dynamics**

In the absence of control measures, mosquito population dynamics are governed by a time-delay differential equation model that is adapted from Dye.^18^ The model explicitly tracks the number of adult females (F) but also accounts for density dependent survival of the larval mosquito stages:

**Figure:** Equation 1

\begin{equation*}\frac{dF}{dt}=PF_{t-\tau}exp^{(-\alpha(EF_{t-\tau})^{\beta})}-dF \end{equation*}

P determines the per capita reproduction rate corrected for density independent mortality prior to adult emergence; E is the daily egg production rate per female and d denotes the adult mortality rate.^18^^,^ 19 ^,^ 20 The time-delay component accounts for the generational time (τ) of *A. aegypti* in the wild (hence, F_t-τ_ equates to the adult female mosquito population τ days ago). α and β respectively govern the carrying capacity of mosquito larvae and the intensity of density dependence. Properties of this form of density dependence are fully explored by Bellows;^21^ but in short, higher β tends towards ‘scramble-type competition’ whereby some of the shared resource will be consumed by all larvae before its limitation imposes additional (density dependent) mortality. Recent field studies exploring *A. aegypti* larval survival provide good evidence to support the fact that this species exhibits strong over-compensatory (scramble type) density dependence.^22^ Baseline model parameterisation used estimates from the range described by Dye (1984): β = 0.571, P = 0.692, d = 0.12 and τ = 27 (see supporting material for further details). However, allowing for ±20% random variability (uniform distribution) in these input parameters, parameter sets were generated that encompassed under-compensatory as well as over-compensatory population dynamics (see Supplementary Figure 1).

**Incorporating control in the mathematical model**

Both the suppression methods and *Wolbachia* operate through released insects mating with wild insects and an important constraint on the efficacy of these approaches is the reduced mating competitiveness of insects reared in the lab versus wild insects. Taking into account the fitness cost associated with lab rearing and denoting the resulting reduction in male competitiveness with c (between 0 and 1), the reduction in reproductive potential of a population under control is

**Figure:** Equation 2

\begin{equation*}\phi =\frac{M_{t-\tau}}{M_{t-\tau}+M_{t-\tau}^{w}+c(M_{t-\tau}^{w,n}+M_{t-\tau}^{s}) }  \end{equation*}

where wild males (M) compete with introduced sterilised males (M^s^) and *Wolbachia*-infected males (M^w,n^), as well as *Wolbachia*-infected males that emerge following wild mating events (M^w^). In the above equation, we assume that the reduced competitiveness associated with lab-reared insects is negligible in their offspring with wildtypes (in other words M^w^ is not multiplied by c).

The recent field trial of RIDL releases in Bahia, Brazil yielded a dataset that included estimates of the density of wild mosquitoes, the density of released males as well as the proportion of RIDL larvae collected in ovitraps that were regularly checked.^12^ Mating competitiveness of lab-reared mosquitoes was tracked over the course of the suppression programme. We explore the relationship between wild female mosquito density and the mating competitiveness of reared male insects using this unique data set.

Because larvae do not hatch from eggs resulting from females mating with radiation sterilised males, this control measures also impacts the intensity of density dependent competition at the mosquito’s larval stage. The proportion of offspring (not infected with *Wolbachia*) that survive to compete at the larval stage is

**Figure:** Equation 3

\begin{equation*}\psi=\frac{M_{t-\tau}+\lambda M_{t-\tau}^{s} }{M_{t-\tau}+M_{t-\tau}^{s}+M_{t-\tau}^{w}+M_{t-\tau}^{w,n}} \end{equation*}

where λ operates as a switch to distinguish between radiation- and genetically-sterilised males: λ = 0 denotes SIT where offspring do not survive to larvae and therefore do not contribute to density dependent competition, and λ = 1 denotes RIDL where RIDL offspring do survive the larval stage.

The resulting population dynamics of female mosquitoes not infected with *Wolbachia* is then

**Figure:** Equation 4

\begin{equation*}\frac{dF}{dt}=PF_{t-\tau}\phi exp^{(-\alpha(\psi EF_{t-\tau})^{\beta})}-dF \end{equation*}

Similarly, the dynamics of *Wolbachia*-infected female mosquitoes is

**Figure:** Equation 5

\begin{equation*}\frac{dF^{w}}{dt}=PF_{t-\tau}^{w}\phi^{w}  exp^{(-\alpha(\psi^{w}  EF_{t-\tau}^{w})^{\beta})}-dF^{w}  \end{equation*}

where the reduction in reproductive potential of the *Wolbachia*-infected population is

**Figure:** Equation 6

\begin{equation*}\phi^{w}=\frac{M_{t-\tau}+M_{t-\tau}^{w}+cM_{t-\tau}^{w,n}}{M_{t-\tau}+M_{t-\tau}^{w}+c(M_{t-\tau}^{w,n}+M_{t-\tau}^{s}) }  \end{equation*}

and the proportion of *Wolbachia*-infected offspring that survive to compete at the larval stage is

**Figure:** Equation 7

\begin{equation*}\psi^{w} =\frac{M_{t-\tau}+\lambda M_{t-\tau}^{s}+M_{t-\tau}^{w}+M_{t-\tau}^{w,n} }{M_{t-\tau}+M_{t-\tau}^{s}+M_{t-\tau}^{w}+M_{t-\tau}^{w,n}} \end{equation*}

ψ, ψ^w^, φ, \begin{equation*}\small{\phi^{w}}\end{equation*}all equal 1 in the absence of control and the original equation is regained.

To be clear, both the SIT and *Wolbachia* simultaneously reduce reproductive potential of wild populations but also increase survival rates at the larval stage through reduced competitive pressure for resources. However, because RIDL offspring survive the larval stage (perishing at the pupal stage), they do not reduce competitive pressure and larval stage survival rates are not increased.

**Simulated field releases of SIT, RIDL and *Wolbachia*-infected mosquitoes**

The three strategies are simulated across a range of mass rearing facility production levels and their efficacies in controlling wild *A. aegypti* populations presented. Field trials of *Wolbachia* have involved weekly releases (allowing for between-release replenishment of mass rearing facility insect stock) for 6-8 weeks;^16^ and RIDL trials have involved slightly more frequent releases (2-3 per week) that have spanned 6 months or more.^12^ For fair comparison, the same release schedule (here, weekly for 3 months) was assumed in simulations for all strategies across a wide range of release ratios (defined as the number of released mosquitoes relative to the wild population pre-control). First, we tested all control methods in isolation for a direct comparison of their efficacies. Then, we simulated integrated control whereby a suppression method (either SIT or RIDL) was used and followed by *Wolbachia* release (smaller populations have previously been shown to enhance *Wolbachia* spread).^23^

## Results

The mating competitiveness of released males relative to wild males appears to be dependent on wild female mosquito density (Supplementary Figure 2). Allowing for this newly identified density dependence, simulated *Wolbachia* invasion occurred quicker for higher release ratios (the ratio of released mosquito numbers relative to wild mosquitoes pre-control) but with diminishing returns: a release ratio of 30 took 150 days for *Wolbachia* to be present in >90% of the population; a release ratio of 100 took 125 days and a ratio of 300 took 120 days (Figure 1; qualitatively identical results were obtained from stochastic simulations allowing for input parameter uncertainty – see Supplementary Figure 3). Importantly, the wild (arbovirus susceptible) mosquitoes exhibited temporary increases beyond pre-control levels during the period before *Wolbachia* could successfully invade (Figure 1; see also Supplementary Figure 3). There is a trade-off between the reduced reproductive potential of wild females and the reduced larval competition pressure following uninfected females breeding with infected males, whereby the former effect is temporarily dominated by the latter. This exacerbation lasted up to 6 weeks and was substantial – at its peak the wild adult female population was doubled.

**Figure:**
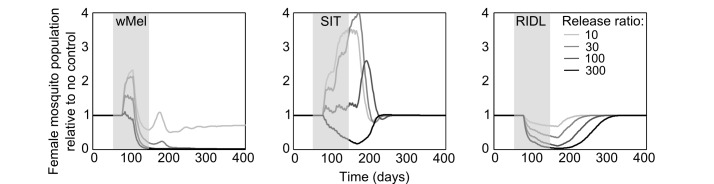
**Fig. 1: Comparative efficacies of three novel methods for *A. aegypti* control** The comparative efficacies in *A. aegypti* control of releasing *Wolbachia*-infected mosquitoes (wMel strain), the Sterile Insect Technique (SIT) and the Release of Insects carrying a Dominant Lethal gene (RIDL). Changes in the potentially infectious (i.e., not infected with *Wolbachia*) female mosquito population during and after control are shown. The thick grey bands correspond with 13 weekly simulated releases. Projections from the underlying deterministic model with fixed baseline parameterisation are displayed here; projections incorporating parameter uncertainty (±20%) give qualitatively identical results and are shown in the supporting material.

Simulations of SIT exhibited a similar but more pronounced exacerbation, lasting up to 140 days with a four-fold higher peak in the wild adult female population relative to no control. However, a release ratio of 300 was sufficient to negate this temporary, undesirable effect (stochastic simulations incorporating uncertainty in model input parameters showed that even for this release ratio, increases in the female vector populations occurred but these increases were both small and short-lived, see Supplementary Figure 3). After 120 days of the initial sterile male release, the wild female population was suppressed by >80%. A similar control efficacy was generated when simulating RIDL at a release ratio of 100 (i.e. one-third the release required for equivalent control by SIT). Three months of RIDL release at a ratio of 300 suppressed the wild adult female population for over 9 months (280 days), with greatest control efficacy of >95% also at 120 days following the initial release. By 160 days after final release of either SIT or RIDL males, the wild population was completely replenished.

Combining the suppression strategies with *Wolbachia* releases was simulated to determine whether the undesirable effect of temporarily exacerbating the wild mosquito population could be dampened or eliminated. Again, for transparent comparison with the standalone strategies, the total number of releases were kept constant (13 weekly releases). Different scenarios of combining SIT with *Wolbachia* demonstrated both potential synergistic and antagonistic effects (Figure 2). Antagonism only arose from attempts to complement *Wolbachia* with SIT release ratios shown to be insufficient to suppress the wild population as a standalone approach. This poor pairing either delayed *Wolbachia* invasion (when a suppression strategy was switched to a *Wolbachia* strategy at week 3, 6 or 9 in a 13-week release schedule), or, at its worst, blocked *Wolbachia* invasion when strategy switching occurred late in the release schedule (week 12; see Supplementary Figure 4 for similar findings from the stochastic model). However, releasing *Wolbachia* after an initial suppression strategy with higher release ratios of SIT males (of 300) produced the highly desirable outcome of successful invasion of *Wolbachia*-infected without the temporary exacerbation in the wild adult female population. In the stochastic model, small and very short-lived exacerbations resulted but these represented considerable reductions when compared with the effects of *Wolbachia* alone (see Supplementary Figure 4). A synergistic result was also achieved by complementing *Wolbachia* with RIDL (Figure 2).

**Figure:**
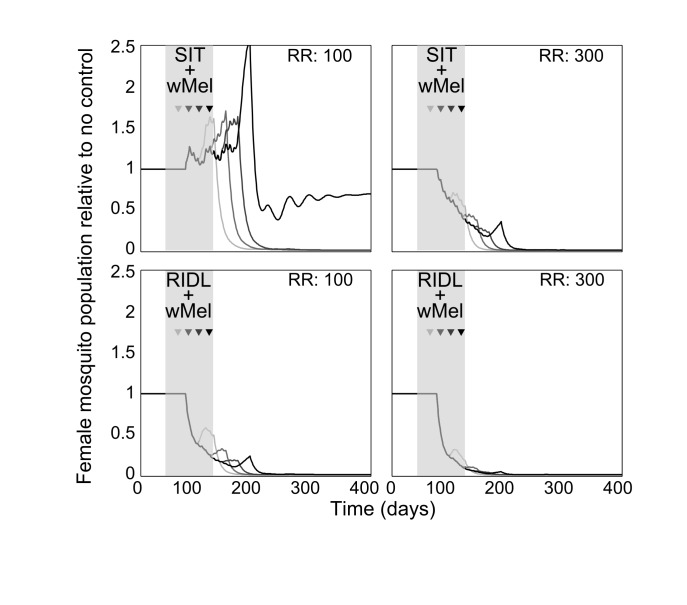
**Fig. 2: Following up *A. aegypti* suppression with *Wolbachia* releases** Following up *A. aegypti* suppression methods with wMel releases. Four different release schedules are shown in each sub-plot whereby the four arrows correspond with switching from a suppression method (either SIT or RIDL) to *Wolbachia* release at week number 3, 6, 9 or 12 (darkening shades of arrow) of a 13-week simulated release programme (thick grey band). The different release schedules project differing control efficacies whereby the colour of lines corresponds to the arrows depicting the release schedule (e.g. the darkest line corresponds with a switch from suppression with either RIDL or SIT to *Wolbachia* during the 12th week of a 13-week control programme). Results are shown for two release ratio scenarios – top right of sub-plots. Projections from the underlying deterministic model with fixed baseline parameterisation are displayed here; projections incorporating parameter uncertainty (±20%) give qualitatively identical results and are shown in the supporting material.

The optimal timing was sought for switching from the suppression strategies to *Wolbachia* release. Extensive simulations were conducted to identify release schedules that evaded transient exacerbation in the wild adult female population (Figure 3). When high release ratios were achievable (>225), inadvertent increases in wild populations were not seen when using SIT-*Wolbachia* across all release schedules. An equivalently safe control was achievable using RIDL-*Wolbachia* at lower release ratios (>115); and avoiding wild population exacerbation became more assured (requiring lower release ratios) with later switching from suppression to *Wolbachia*. The trade-off with switching strategies later in the release schedule is that it might delay *Wolbachia* invasion: at a release ratio of 250, a switch at the mid-point (week 7) compared to week 2, delays *Wolbachia* invasion by 30 and 20 days for SIT and RIDL, respectively. However, at lower release ratios (25-140 for SIT and 15-30 for RIDL), the timing of the strategy switch can make the difference between success and failure in *Wolbachia* invasion.

**Figure:**
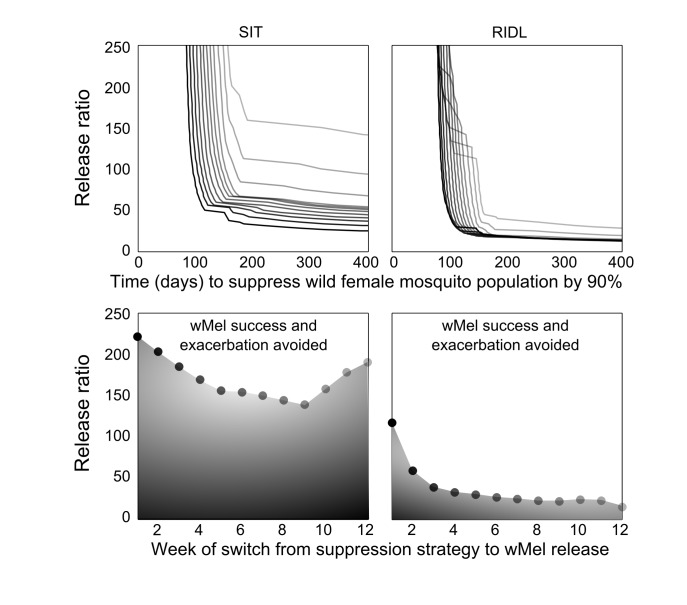
**Fig. 3: Optimizing the modernized, integrated vector management** Top: Lower release numbers delay or preclude the invasion of wMel following SIT (left column) or RIDL (right column). Darker lines represent an earlier timed switch from suppression to *Wolbachia* release (corresponding with the markers in the lower sub-plots). Bottom: Timing of the switch from suppression to wMel affects the release ratio required to negate temporarily increasing wild *A. aegypti* populations (relative to no control).

## Discussion

In the wake of the largest Zika virus outbreak in history, there have been calls for a paradigm shift towards a more modern strategy for controlling vectors.^6^^,^ 24 Several novel *A. aegypti* control methods, namely SIT, RIDL and the release of* Wolbachia*-infected mosquitoes, have recently been endorsed by the WHO for use in the containment of the ongoing Zika virus outbreak.^17^ The current study sought to provide an initial, comparative assessment of these tools as well as to strategize their use as part of a modernized, integrated vector management programme.

In simulating a release schedule designed to emulate those of recent *Wolbachia* 16 and RIDL^12^ field trials, the novel tools demonstrated important pitfalls and opportunities in controlling *A. aegypti* populations. The invasive nature of *Wolbachia* means that, provided a sufficient number of *Wolbachia*-carrying mosquitoes are released, production of an arbovirus-resistant mosquito population is sustained – and this has been shown by previous assessments, both theoretical^23^ and empirical.^25^ However, due to the intense and over-compensatory density dependence in the mosquito’s larval stages,^18^^,^ 22 the initially small reduction is more than offset by the alleviated pre-adult competition. This means that a temporary increase in the wild mosquito population may result. This undesirable effect is mirrored by simulations of SIT which also results in eggs that do not hatch and thereby alleviates competition pressure at the larval stages. However, using sufficiently high release ratios that were still within the range achieved by recent field trials of sterilised males, this exacerbation could be avoided (or, at least, significantly attenuated) in an SIT programme.

Provided sufficiently high numbers of mosquitoes were released and the timing of switching strategies was appropriate, the suppression capabilities of SIT and RIDL proved useful in negating the undesirable, temporary increase in wild *A. aegypti* populations following *Wolbachia* release. To the best of our knowledge, this represents the first theoretical evidence to support combining these strategies.

The current analysis has important limitations. A simple underlying model was selected to present as transparent a comparison as possible between the three control methods. Simple models are most useful during early stages of strategy development; the current study is not intended for the development of specific operational tactics and should not be interpreted as such. Work in progress includes a comprehensive sensitivity analysis to identify if and when qualitative shifts occur in strategic recommendations with seasonally fluctuating mosquito populations; and, a more detailed representation of the strategies which will include additional fitness costs associated with either radiation exposure, transgene insertion or *Wolbachia* infection as well as incomplete sterility and imperfect *Wolbachia* heritability.^11^^,^ 13 ^,^ 25 ^,^ 26 Additionally, major modelling advances have been made in recent years including a Bayesian hierarchical model for estimating spatial mosquito density;^27^ and detailed life history models of *A. aegypti* that include temperature-dependent development rates,^28^^,^ 29 ^,^ 30 as well as mosquito movement and spatially explicit larval habitats.^31^ These should be capitalised upon to accelerate corresponding developments in the current context. Finally, potential synergisms should be sought between these modern methods to vector control and more traditional methods including larval site destruction and chemical insecticides. Beyond this, an epidemiological component should be incorporated to ascertain the impacts of these methods on disease transmission dynamics - the relationship between vector density and disease transmission intensity is not clear-cut and requires further research.

Despite best efforts, the global distribution of *A. aegypti* is rapidly expanding along with the numbers of countries reporting arboviral infections.^32^^,^ 33 If this trend is to be halted or reversed, a new framework will be needed to inform optimal control strategy that reduces dependence on mosquito management practices that are known to have dwindling efficacy, and that takes advantage of the increasing number of novel methods becoming available.

## Competing Interests

The authors have declared that no competing interests exist.

## Corresponding Author

Laith Yakob: laith.yakob@lshtm.ac.uk

## Data Availability

All relevant data are openly accessible and referenced within the paper.

## Appendix

**Figure:**
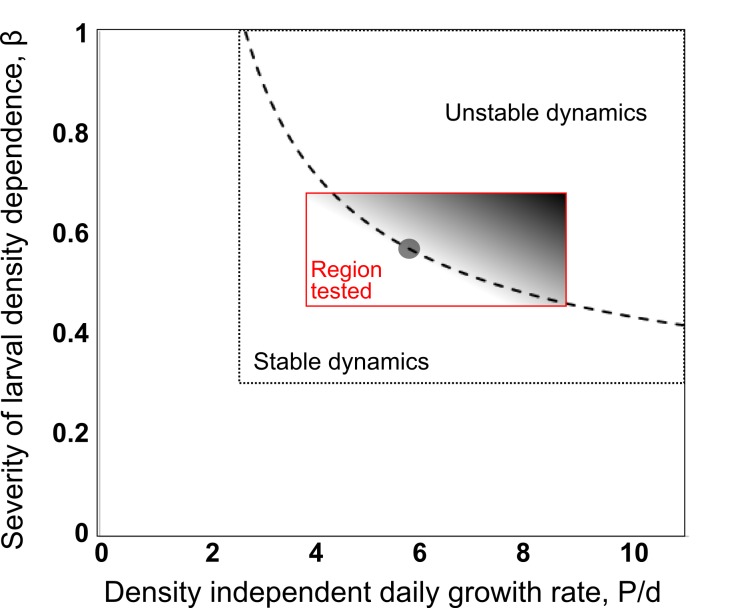
**Supplemental Fig. 1: Stability regions for an* Aedes aegypti* population**. Quasi-cyclic behaviour (unstable dynamics) occurs when β×ln(P/ d)>1 where β is a measure of severity of the density-dependent response; P is the maximum per capita daily egg production rate corrected for density-independent egg to adult survival, and d is the per capita daily adult death rate (hence P/d is equivalent to the per capita, density independent, daily growth rate). The range of parameter values encompassed by the black box are taken from Dye (1984). The red box denotes the parameter space explored through simulation by allowing β, P and d to all vary by ±20%.

**Figure:**
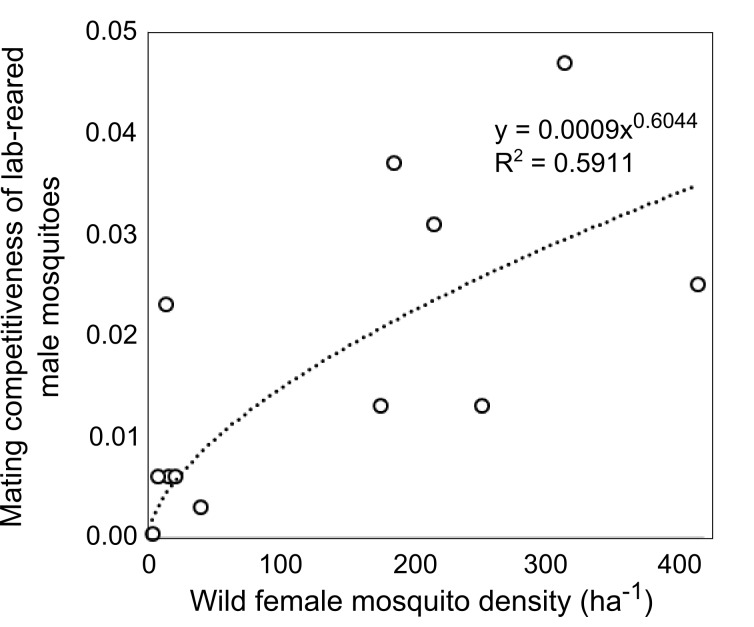
**Supplementary Fig. 2: Mating competitiveness of lab-reared male mosquitoes. **Mating competitiveness of lab-reared male mosquitoes is dependent on the density of wild females. Re-analysis of field data reported by Carvalho et al. (2015).

**Figure:**
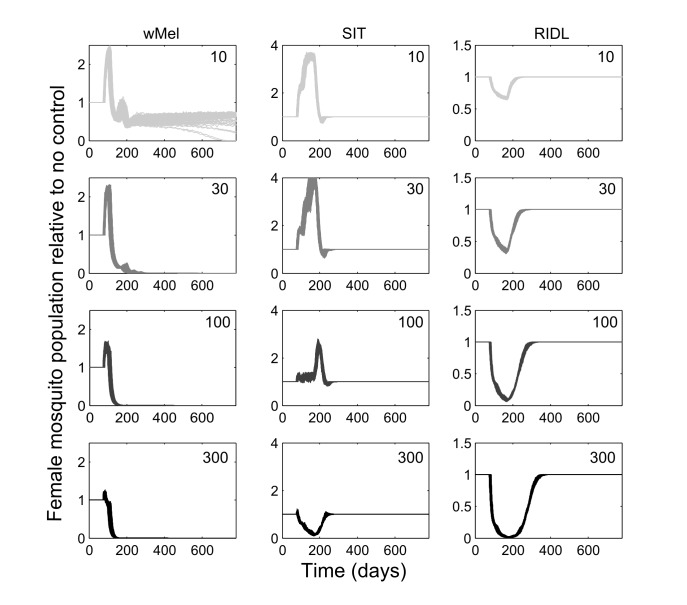
**Supplementary Fig. 3: Comparative efficacies of three novel methods for *A. aegypti* control (stochastic model).** *Aedes aegypti* control with standalone novel technologies (stochastic model). The comparative efficacies in *A. aegypti* control of releasing *Wolbachia*-infected mosquitoes (wMel strain), the Sterile Insect Technique (SIT) and the Release of Insects carrying a Dominant Lethal gene (RIDL). Changes in the potentially infectious (i.e., not infected with *Wolbachia*) female mosquito population during and after control are shown. 100 projections incorporating parameter uncertainty (random variation of ±20% following a uniform distribution for parameters β, P, d and τ) give qualitatively identical results to the underlying deterministic model with fixed baseline parameterisation displayed in the main text. The exception being rare events of successful* Wolbachia* invasion at the low release ratio of 10 (top left sub-plot).

**Figure:**
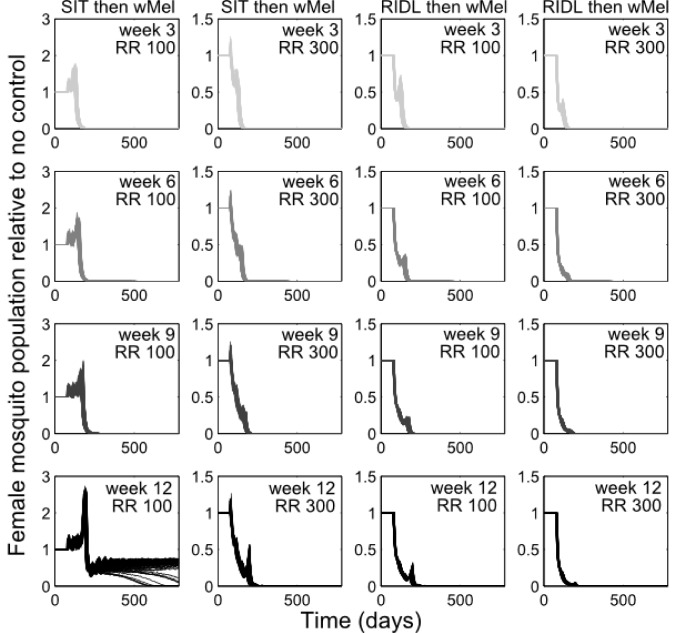
**Supplementary Fig. 4: Following up *A. aegypti* suppression with *Wolbachia* releases (stochastic model).** *Aedes aegypti* control with integrated novel technologies (stochastic model). Following up *A. aegypti* suppression methods with wMel releases. Four different release schedules are shown in the sub-plots switching from a suppression method (either SIT or RIDL) to *Wolbachia* release at week number 3, 6, 9 or 12 of a 13-week simulated release programme. Results are shown for two release ratios (‘RR’ of 100 and 300) – top right of sub-plots. 100 projections incorporating parameter uncertainty (random variation of ±20% following a uniform distribution for parameters β, P, d and τ) give qualitatively identical results to the underlying deterministic model with fixed baseline parameterisation displayed in the main text. The exception being relative rare events of successful *Wolbachia* invasion even at the lower release ratio of 100 when following SIT (bottom left sub-plot).
